# Electronic Implementation of a Repressilator with Quorum Sensing Feedback

**DOI:** 10.1371/journal.pone.0062997

**Published:** 2013-05-02

**Authors:** Edward H. Hellen, Syamal K. Dana, Boris Zhurov, Evgeny Volkov

**Affiliations:** 1 Department of Physics & Astronomy, University of North Carolina Greensboro, Greensboro, North Carolina, United States of America; 2 CSIR-Indian Institute of Chemical Biology, Kolkata, India; 3 Department of Theoretical Physics, Lebedev Physical Institute, Moscow, Russia; Universitat Politecnica de Catalunya, Spain

## Abstract

We investigate the dynamics of a synthetic genetic repressilator with quorum sensing feedback. In a basic genetic ring oscillator network in which three genes inhibit each other in unidirectional manner, an additional quorum sensing feedback loop stimulates the activity of a chosen gene providing competition between inhibitory and stimulatory activities localized in that gene. Numerical simulations show several interesting dynamics, multi-stability of limit cycle with stable steady-state, multi-stability of different stable steady-states, limit cycle with period-doubling and reverse period-doubling, and infinite period bifurcation transitions for both increasing and decreasing strength of quorum sensing feedback. We design an electronic analog of the repressilator with quorum sensing feedback and reproduce, in experiment, the numerically predicted dynamical features of the system. Noise amplification near infinite period bifurcation is also observed. An important feature of the electronic design is the accessibility and control of the important system parameters.

## Introduction

Biological networks such as transcriptional genetic networks and metabolic networks are complex in structure and dynamical behaviors [1]. A systematic understanding of the functional behaviors of such networks is challenging both from theoretical and experimental viewpoints. Alternatively, an engineering approach [2]–[4] has been undertaken, in recent times, to simulate desired biological functions by designing small genetic units. The main target is to integrate large numbers of such units to derive complex biological functions [5]–[7]. This is like engineering small integrated chips to build a computer to perform a desired function. One of the important discoveries, in this direction, is the design of an artificial genetic network known as a repressilator [4] which consists of a ring of three genes inhibiting each other in cyclic order that shows oscillatory behaviors. Theoretical studies of the single deterministic, stochastic, and even electronic repressilator have attracted attention of many investigators [8]–[17].

On the other hand, quorum sensing (QS) [18], [19] is a typical process of communication in a bacterial colony, and it has been used as a mechanism for coupling synthetic genetic oscillators [20]–[25]. The QS is accomplished by diffusive exchange of small auto-inducer (AI) molecules which participate in the intercellular coupling as well as in self-feedback. Recently, the effective diffusion of AI has been demonstrated experimentally to induce coherence of oscillation in a small colony of synthetic gene units [25]. It is important to understand the effect of the AI feedback on a single repressilator before considering the design of a network of multiple units. We focus on the effect of the self-feedback that QS may provide to a single isolated repressilator and try to understand its dynamics. Details of the model are reported recently [26].

Here, we report a variety of interesting dynamics in the isolated repressilator with a QS type feedback such as multi-stability of limit cycle with stable steady-state (SS), multi-stability of different stable steady-states, limit cycle (LC) with period-doubling (PD) and reverse period-doubling (RPD) and infinite period bifurcation (IPB) transitions for both increasing and decreasing strength of quorum sensing feedback. A noise amplification near the IPB is also observed. Our results are based on both numerical simulation and experimental measurement. Experiments are accomplished by designing an electronic circuit analog of the repressilator with QS feedback. The electronic design is carefully made so that the key system parameters are accessible and controllable, which is not easy in real biological experiments. The electronic analog of the synthetic genetic network is thereby able to reproduce the important dynamical features predicted numerically. The electronic bench-work provides an actual physical system, including the presence of intrinsic system noise, external noise, and device mismatch and, also produces results free of numerical artifact. As such, we use both numerical simulations and circuit measurements as complementary approaches for investigating the dynamics of the particular network topology. This knowledge about the dynamics of the network may be used later for planned biological experiments.

We present the model and the numerical results first, followed by the electronic circuit and experimental measurements. Details of the circuit analysis and derivation of the relation between circuit parameters and model parameters are given in Appendix S1.

## Model and Numerical Results

**Figure 1:**
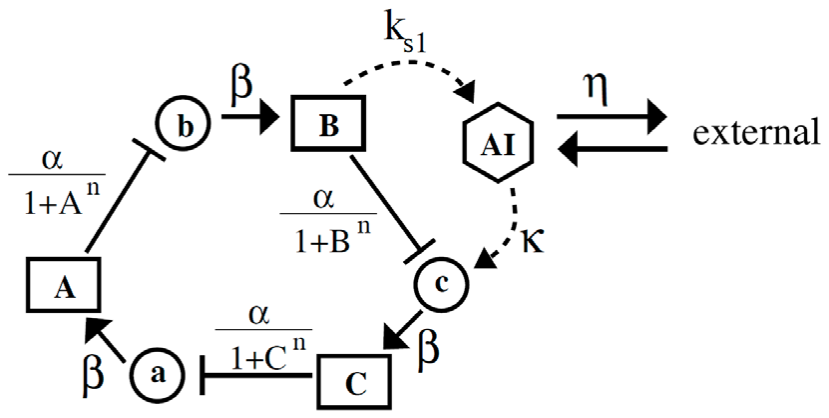
Repressilator genetic network with QS feedback. Lower case (*a*, *b*, *c*) are *m*RNA and upper case (*A*, *B*, *C*) are expressed protein repressors. AI is the auto-inducer molecule which activates production of protein *C* and also diffuses through the cell membrane.

We first investigate the dynamics of a single repressilator with QS feedback using numerical tools. The starting point is the repressilator with QS feedback [15] as shown in Fig. 1. The three genes in the loop produce mRNA (*a*, *b*, *c*) and proteins (*A*, *B*, *C*), and they inhibit each other in cyclic order by the preceding gene. The QS feedback is maintained by the AI produced (rate *k*_S1_) by the protein *B* while the AI communicates with the external environment and activates (rate *κ*) production of protein *C* which, in turn, reduces the concentration of protein *A* resulting in activation of protein *B* production. The protein *B* plays a dual role of direct inhibition of protein *C* synthesis and AI-dependent activation of protein *C* synthesis, resulting in complex dynamics of the repressilator.

The original model of a repressilator [4], [15] used re-scaled dimensionless quantities for rate constants and concentrations. We reduce the model for the case of fast mRNA kinetics (*a*, *b* and *c* are assumed in steady state with their respective inhibitors *C*, *A*, *B*, so that *da*/*dt* = *db*/*dt* = *dc*/*dt* ≈ 0). The resulting equations for the protein concentrations and AI concentration *S* are,
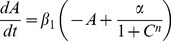
(1a)

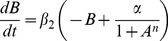
(1b)

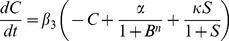
(1c)

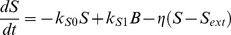
(1d)where the *β*_i_ is the ratio of protein decay rate to mRNA decay rate, *α* accounts for the maximum transcription rate in the absence of an inhibitor, *n* is the Hill coefficient for inhibition, and *k*_S0_ is the ratio of AI decay rate to mRNA decay rate. The diffusion coefficient *η* depends on the permeability of the membrane to the AI molecule. *S*_ext_ is the concentration of AI in the external medium which is taken to be zero in our case of a single isolated repressilator. We choose parameter values similar to previously used values shown to be experimentally reasonable taking into account realistic biochemical rates and binding affinities [4], [15], [23]. Here we use β = 0.1 to 1, *n* = 2 to 4, *k*_S0_ = 1, *k*_S1_ = 0.025, and *η* = 1. We explore a wide range of values for rates α and κ.

The strength of QS feedback is determined mainly by the activation rate parameter κ. Recently, it has been shown [17] that the dynamics of coupled repressilators depend strongly on the time scale ratio *β*. Therefore we investigate how the dynamics of a single repressilator with QS feedback depends on κ for a small value *β* = 0.1, which is close to biologically motivated value, and for the larger value *β* = 1 which assumes the same time scales for both the proteins and the mRNA. A β-value near 1 was realized in the biological repressilator [4].

### Numerical Results

**Figure 2:**
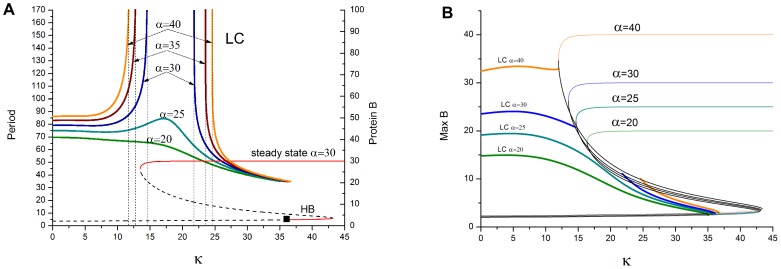
Numerical repressilator bifurcation diagrams as a function of QS strength κ. Shows protein *B* dynamics for *n* = 3.3, *β* = 0.1, *k*_S1_ = 0.025, *k*_S0_ = 1, and η = 1. Note that LC is always period-1 for *β* = 0.1. (a) LC period for various α (left axis). Steady-state protein *B* concentration (red-stable, dashed-unstable) for α = 30 (right axis). LC is continuous to HB for small α (≤25), but has break in continuity for larger α. (b) Protein *B* amplitudes for LC (thick colors, left and lower portion of graph) and SS (thin colors, right and upper portion) for various α. Note that α = 20, 25 have smooth decrease in LC amplitude for increasing κ, whereas α = 30, 40 have sharp transitions to high-*B*-SS due to IPB near κ = 15. HB transition from LC to low-*B*-SS occurs near κ = 36. Low-*B*-SS is lower line from κ = 36 to 43.

We first present the numerical results of available dynamical regimes as stable steady state (SS) and limit cycle (LC) as calculated from Eq.(1) using the XPPAUTO [27] for *β*_1_ = *β*_2_ = *β*_3_ = *β = *0.1 (slow protein kinetics) and *n* = 3.3. Figure 2a shows the period of LC states under different transcription rates α, as well as the protein *B* concentration of SS for α = 30, both as functions of κ. For the α = 30 LC, increasing κ from 0 causes the activation of protein *C* production to become so large that a stable SS with high concentration of protein *B* emerges (upper red line) near κ = 14. This high-*B*-SS coexists with the stable LC over a broad interval of larger κ, from about 22 to 36. For κ in the range 15 to 22 there is a break in LC continuity where the LC is unstable. Near κ = 36 the LC transitions via Andronov-Hopf bifurcation (HB) to a stable SS with low concentration of *B* (lower red line in Fig. 2a). This second SS is stable over a small range of κ (36 to 43) in which it coexists with the high-*B*-SS, thus behaving as a switch. The operation of the 3-gene ring as a bistable genetic switch over this range of κ may be of practical interest. At higher κ (>43), only the high-*B*-SS is stable.

Figure 2b shows protein *B* dynamics, LC amplitude (thick colored lines, left and lower portion of graph) and SS level (thin colored lines, upper and right), for different transcription rates α as a function of κ. For each α the strong dependence of the LC amplitude on κ suppresses oscillations resulting in the HB to the low-*B*-SS near κ = 36. The low-*B*-SS level is about 2.5 for all α, whereas the high-*B*-SS level is approximately α as predicted by Eq. (1b) since protein *A* is small. For small *α* (≤25) the κ-dependence of LC is continuous to the HB in Figs. 2a and 2b because of the low amplitude of LC, but for larger *α* (≥30) the LC becomes unstable and transitions via IPB to the high-*B*-SS because the larger α has larger amplitude oscillations which allow the phase point to reach the SS. Hysteresis is predicted in Figs. 2a and 2b by the overlapping κ-range of the LC and SS for *α = *30. The steeply increasing periods in Fig. 2a indicate the formation of the infinite period regimes between two values of κ for α ≥30. IPBs are realized by increasing κ from small values as well as by decreasing it from large values. The mechanism of this phenomenon in 4D space deserves separate investigation.

**Figure 3:**
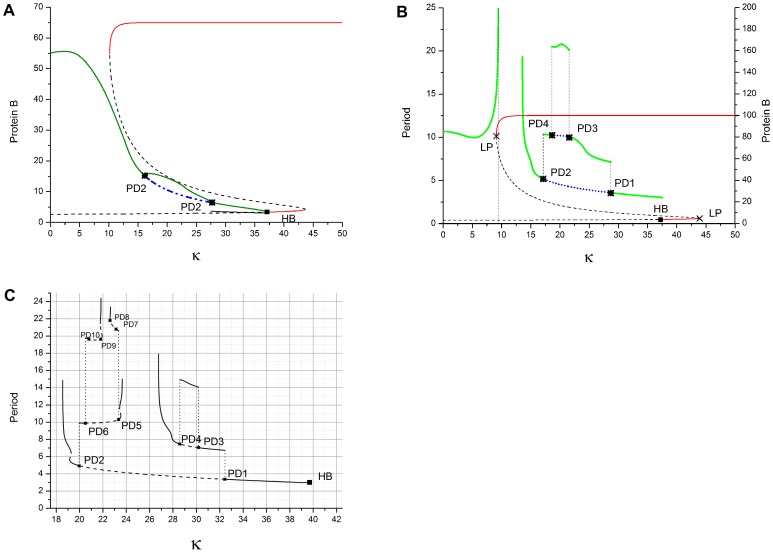
Numerical repressilator bifurcation diagrams as a function of QS strength κ. Shows protein *B* dynamics for *β_i_ = *1, *k*_S1_ = 0.025, *k*_S0_ = 1, and η = 1. Note the increasingly complex dynamics as α increases in frames (a) to (b) to (c). (a) α = 64, *n* = 3.3. SS (red – stable, dashed – unstable) and LC [green – stable period-1 and period-2, blue – unstable period-1]. Note the period doubling of LC to period-2. (b) α = 100 n = 3.3 causes break in LC continuity and more complex PD/RPD. (c) α = 200, n = 3.43. LC (solid – stable, dashed – unstable).

Next we consider *β* = 1 (protein kinetics same as mRNA and AI). The behavior of SS is not affected by this choice but the evolution of the LC is changed dramatically. Figure 3 shows predictions of LC dynamics for various α as κ increases. When α is less than 47 (results not shown here), the LC continuation is continuous period-1 to the HB. However, for larger α the dynamics become more complex. For α = 64 Fig. 3a shows that the LC continuation (green curve) remains stable over the κ interval but is more complex due to the appearance of PD followed by RPD bifurcations. Increasing κ beyond the HB causes a transition to SS (lower red curve) with all proteins low which coexists with the high-*B*-SS creating a genetic bistable switch as occurred for β = 0.1. Further increase of κ causes a jump to the high-*B*-SS (upper red curve). For larger α (Fig. 3b) a break in continuity of LC appears, in addition to more complex PD/RPD cascades. For α = 200, n = 3.43 (Fig. 3c) a second break in LC continuation occurs.

**Figure 4:**
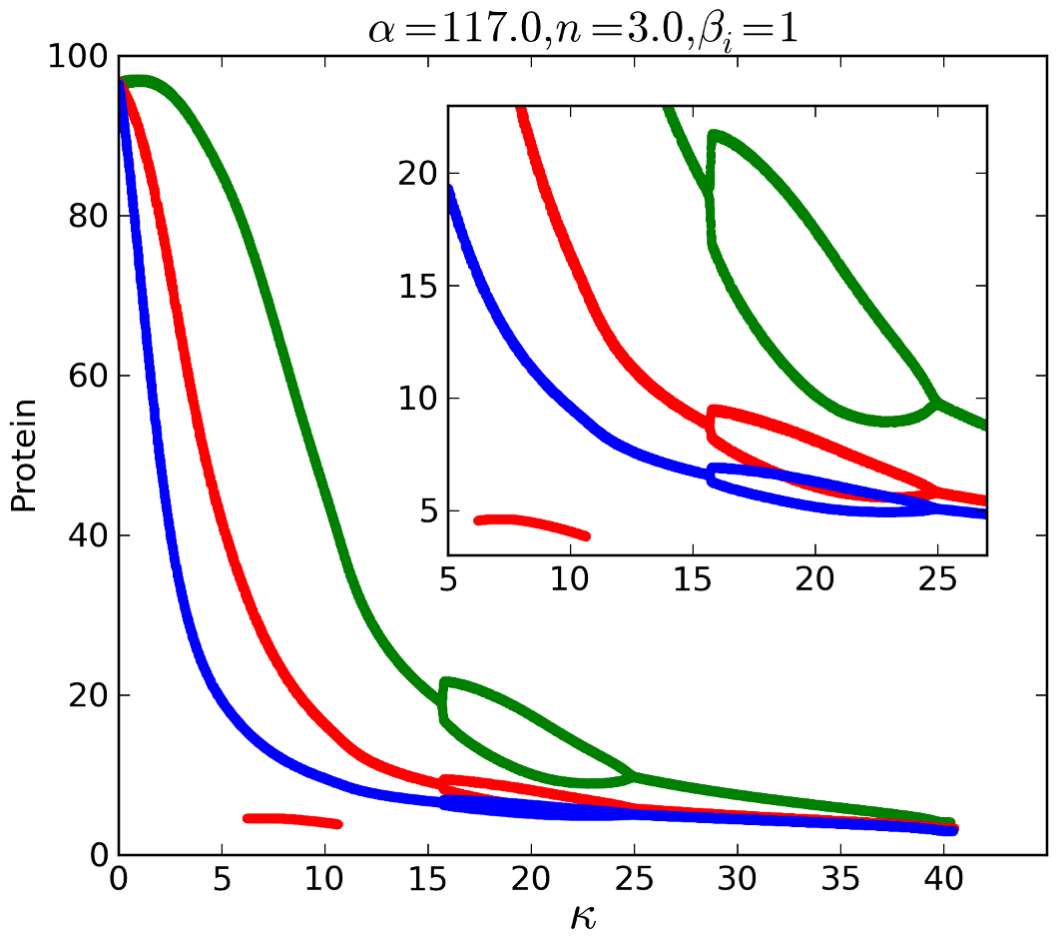
Numerical repressilator bifurcation diagram as a function of QS strength κ. Shows LC local maxima for all 3 proteins for β = 1, *n* = 3, α = 117, *k*_S1_ = 0.025, *k*_S0_ = 1, and η = 1. LC is period-1 for κ = 0 to 16, period-2 for κ = 16 to 25, and period-1 for κ = 25 to HB near 40. Protein *A* (blue), *B* (green), *C* (red).

Figure 4 shows details of how the LC amplitude changes for all three proteins as κ increases for the case β = 1, α = 117, n = 3. The amplitudes of period-1 LCs decrease sharply as κ goes from 0 to 15. Bifurcations to period-2 occur at κ = 16 and back to period-1 at κ = 25, followed by decreasing LC amplitude to SS via HB at κ = 40.

Here we summarize the numerical findings. Increasing the strength of QS feedback has two main effects on the repressilator dynamics. Firstly, when the feedback is increased beyond a critical value, a stable steady state (SS) appears with protein *B* (which controls the AI production) concentration becoming high and the other two protein concentrations becoming low. Multistability exists here between SS and LC. Secondly, as the feedback is further increased, the LC evolves (possibly through unstable regions) with shrinking amplitude until it converges via HB to a different SS characterized by low concentration of all three proteins. Interestingly, the existence of this second SS was previously unknown and allows multistability between the two different SS. The particular dynamics of the evolution of the LC depends on the parameter values. Large transcription rates cause the LC to become unstable for a specific range of QS feedback due to the appearance of IPBs from a LC to a SS. These IPBs occur for both increasing feedback strength from low values and for decreasing feedback strength from high values. A hysteresis between the LC and the SS is seen. A faster kinetics (β = 0.1) of mRNA and QS signaling molecules compared to repressilator protein results simply in period-1 LC dynamics. However, when the QS autoinducer and repressilator proteins have similar time scales (β = 1), then the LC may have complex dynamical behavior which is characterized by the appearance of PD and RPD.

## Electronic Circuit and Measurements

Next we report the circuit realization of the genetic network with quorum sensing feedback. Previously we reported an electronic version of the repressilator (e-Rep) [28] which consists of three genes coupled in a ring, inhibiting each other in a cyclic order. The strength of this electronic analog is its accuracy in reproducing the Hill function nonlinearity that controls the rate of transcription. The circuit allows an effective control of the system parameters. As a result, it reproduced the basic properties of an isolated repressilator. Here we make further improvement upon the Hill function nonlinearity in the circuit design and include additional circuitry to imitate the QS feedback. In this way we study how the addition of QS feedback to an isolated e-Rep changes its dynamical behavior and we verify the numerical results. The circuit uses the reduced 4-dimensional model (Eq. 1) of the repressilator in which only three equations for protein dynamics and one for AI are simulated since the mRNA kinetics are assumed to be fast enough to keep mRNA in quasi-equilibrium with its inhibitor.

One can couple many of the e-Rep to simulate various desired functional behaviors thus enabling prediction or design of collective behaviors quantitatively by controlling the circuit parameters analogous to genetic network’s parameters. The predicted behaviors can be used for decisive biological experiments. This is a major benefit in studies of e-Rep circuits over the biological experiments where *a priori* knowledge of the system parameters is not available. The control of the system parameters is a very difficult task in a biological experiment which has additional complications due to its fluctuating medium. We can include additive or multiplicative noise in e-Rep experiments to imitate some types of the fluctuations encountered in real biological experiments. Another target of designing e-Rep is to design new electronic devices (e.g., logic gate, switch) [29] based on the knowledge of synthetic genetic networks.

**Figure 5:**
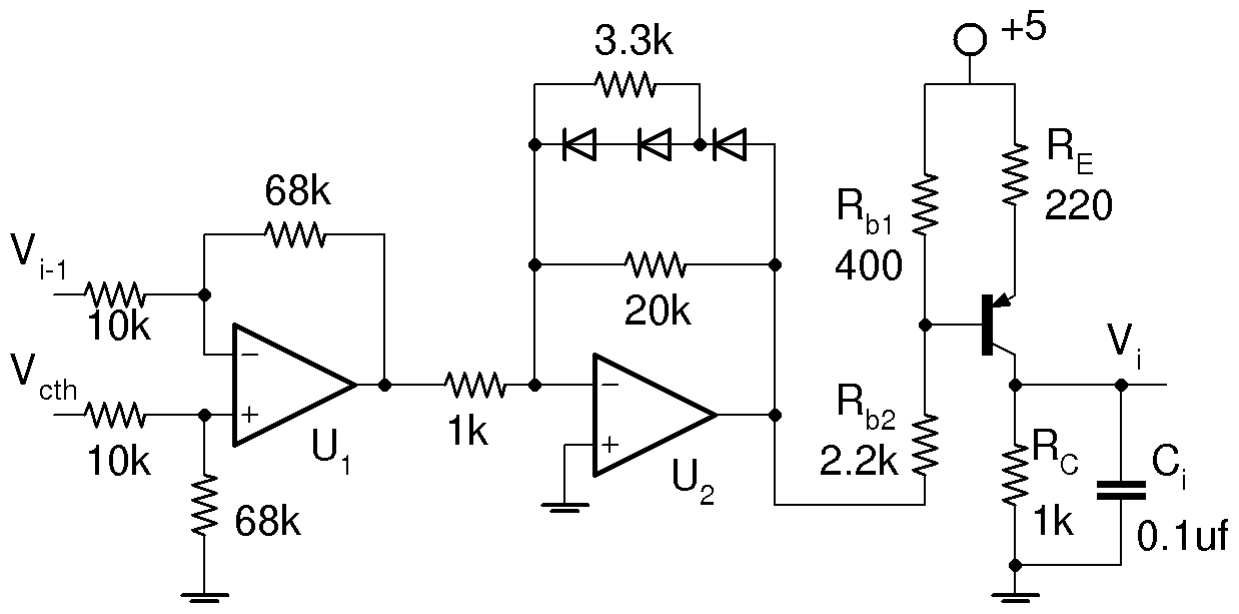
Electronic analog of a single gene with inhibitory input. *V*_i−1_ is proportional to the concentration of repressor and *V*_i_ is proportional to the concentration of expressed protein. *V*_cth_ accounts for the binding affinity of the repressor to the gene DNA. When *V*_i−1_<< *V*_cth_ then the output of U_2_ is low and the transistor supplies maximum current (no inhibition). When V_i−1_>> V_cth_ then the transistor is shut off (complete inhibition). U_1,2_ are LF412 op-amps supplied by ±5-volts, *pnp* is 2N3906, diodes are 1N4148. The diodes in feedback produce the Hill function inhibition kinetics.

We implement the model (Fig. 1) in an electronic circuit based on the e-Rep circuit used in Ref. 28. Figure 5 shows the electronic analog of a single gene used here, which is a slight modification of the circuit used in Ref. 28 with an improved Hill function nonlinearity. The relation between the circuit components and the model parameters *n*, *α*, *β*, *κ*, *k*_S0_, *k*_S1_, *η* are given below. We use *k*_S0_ = 1, *k*_S1_ = 0.025, and *η = *1, in this paper.

**Figure 6:**
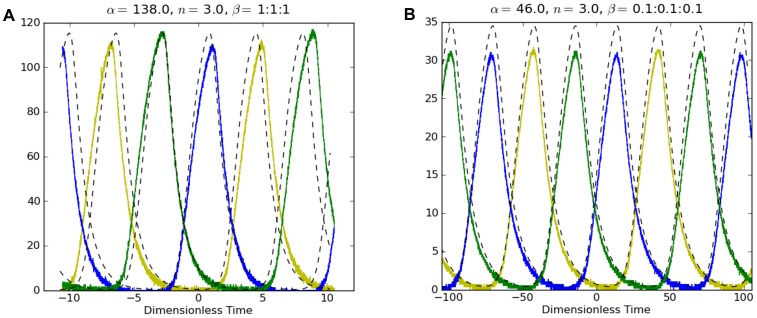
Simulated (dashed lines) and experimental time series (solid blue, green and yellow lines) for single e-Rep without QS. (a) β_i_ = 1, (b) β_i_ = 0.1. Ordinates are the normalized protein concentrations *A, B, C*.

The equation for the voltage *V_i_* across the capacitor *C*_i_ in Fig. 5 is obtained by using the Kirchhoff’s laws,
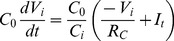
(2)where *I*_t_ is the transistor current which depends on the *inhibitory* input *V*_i−1_. The ratio *V*_i_/*V*_th_ is the normalized state variable for protein concentration (*i* = *A*,*B*,*C*). The relation between the model parameters (*α*, *β*) and the circuit parameters are already derived [28] as reproduced here, *α* = *I_max_R_C_*/*V_th_* and *β*_i_ = *C_0_*/*C_i_* where *C*_0_ defines the dimensionless time *t*/(*R*_C_ *C*_0_). The Hill coefficient *n* and the parameters *V_th_* and *V_cth_* are redefined here as affected by the modification of the circuit in Ref. 28. In Appendix S1, we show that *n* comes from *nα = *2.35*G_1_G*_−*2*_ (compared to 1.5*G_1_G*_−*2*_ in Ref. 28) where *G*_1_ and *G*_−2_ are unsaturated gains of op-amps U_1_ and U_2_, maximum transistor current *I_max_* ≈ 3 mA, maximum *V*_i_ is *I_max_R_C_* = 2.7 V, and *V*_cth_ ≈ *V*_th_ +1/(*G_1_G*_−*2*_). Numerically simulated and experimental oscillations of the 3-gene e-Rep without QS feedback for *β_i_* = (0.1, 1) are shown in Fig. 6 for comparison.

### Circuit with Quorum Sensing

**Figure 7:**
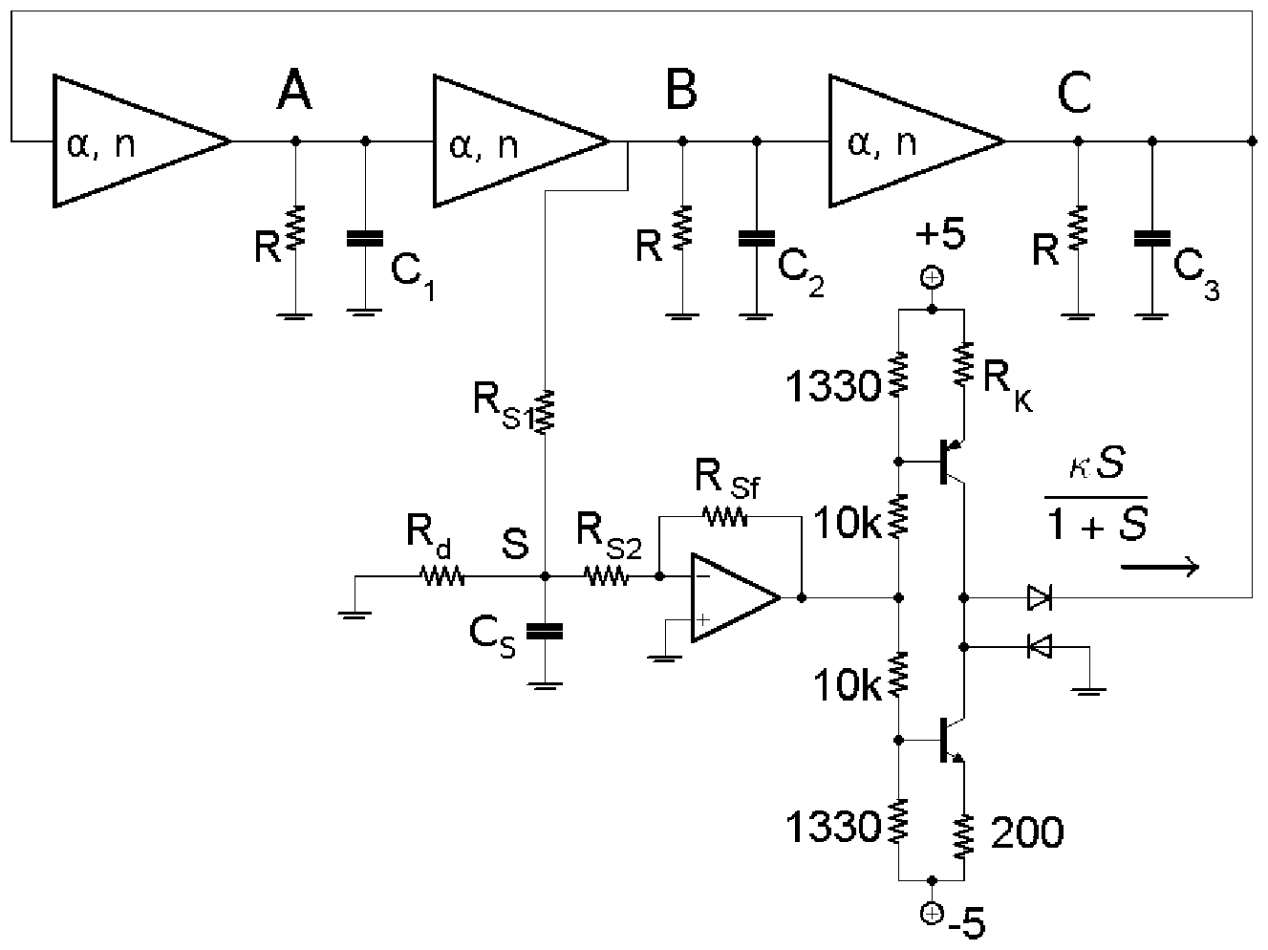
Isolated e-Rep circuit with quorum sensing feedback. Protein *B* stimulates production of auto-inducer *S* that activates production of *C*. When the voltage at *S* increases, the inverting op-amp output drops, which turns the *pnp* transistor on and the *npn* off, thus sending current to protein *C* voltage. Each triangle with parameters *α*, *n* and subsequent *R* and *C*_i_ represents the single-gene circuit in Fig. 5. Resistor R_d_ connected to ground indicates the e-Rep is isolated corresponding to AI concentration outside of the cell being *S*_ext_ = 0. Transistors are 2N3904 (*npn*) and 2N3906 (*pnp*).



Figure 7 shows the circuit for an isolated e-Rep with QS. The triangular blocks with (α,*n*) and subsequent R and C_i_ are the individual genes of Fig. 5. Details of the QS feedback loop show the connections between protein concentrations *B* and *C* corresponding to the feedback loop between *B* and *c* in Fig. 1. The equation for the voltage *V_S_* across *C*_S_ corresponding to the AI concentration *S* is
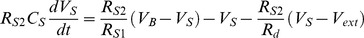
(3)where V_ext_ = 0 since R_d_ is connected to ground in Fig. 7 indicating an isolated e-Rep. Choosing R_S2_C_S_ equal to the characteristic time R_C_C_0_ (which sets k_S0_ = 1) and normalizing by V_sth_, and then using S = V_S_/V_sth_, Eq. (3) is converted,
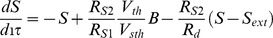
(4)where τ is a dimensionless time. We choose the component values such that [1– RS2Vth/(RS1Vsth)] ≈ 1 since [RS2Vth/(RS1Vsth) <<1 )]. The model parameters (kS1, η) are now expressed in terms of the circuit parameters,

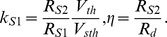



We use *R*_S1_ = 100 kΩ and *R*_S2_ = 6.8 kΩ in the circuit. For a single e-Rep with QS feedback, *S*_ext_ = *V*_ext_/*V*_sth_ = 0.

In Appendix S1 we show that, 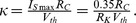


The circuit approximates the *S*-function *S*/(1+*S*) by a piecewise linear function, *min*(S/(1+S_cr_),1) where *S*_cr_ is the value of *S* where the piecewise linear function crosses *S*/(1+*S*). We typically choose *S*_cr_ = 0.25. We show that *S*_cr_ determines [see Appendix S1] the feedback resistor *R*_Sf_ by
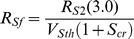



### Circuit Measurements

**Figure 8:**
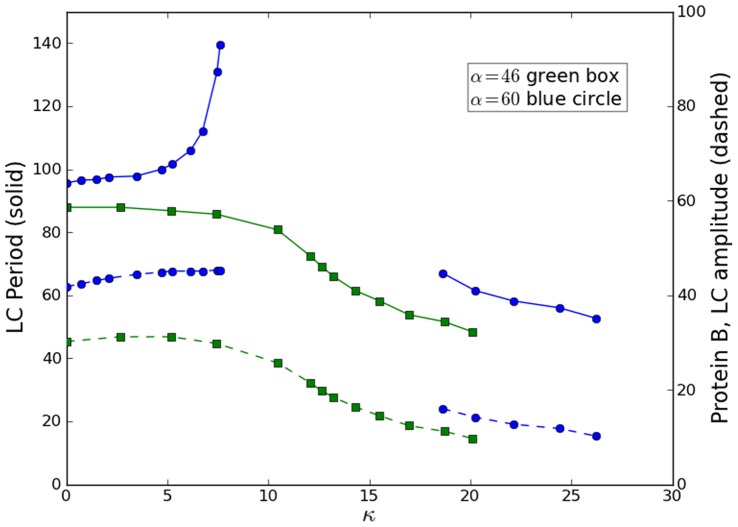
Experimental e-Rep bifurcation diagrams as a function of QS strength κ. Shows measured LC periods (solid lines, left axis) and protein *B* amplitudes (dashed, right axis) for α = 46 (green), 60 (blue). Other parameters are *n* = 3.0, β = 0.1, *k*_S1_ = 0.025, *k*_S0_ = 1, and η = 1. For α = 60 there is a break in κ-continuity of LC caused by IPB to SS.

**Figure 9:**
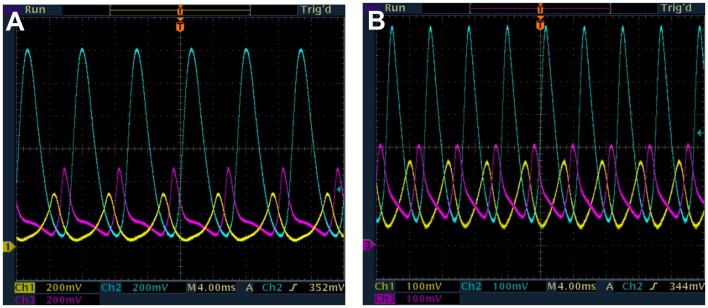
Measured time series from e-Rep circuit showing the period-1 LC (protein *A*-yellow, *B*-blue and *C*-purple). β = 0.1, α = 46, n = 3, *k*_S1_ = 0.025, *k*_S0_ = 1, and η = 1. (a) κ = 14, (b) κ = 19. Note that the protein oscillation amplitudes are larger in frame (a) than (b) since the vertical scale is 200 mV/div in (a) and 100 mV/div in (b). Thus the decrease in both period and amplitude with increasing QS interaction is apparent.

Now we present measurements from the e-Rep with QS feedback. Numerical predictions in Fig. 2 for *n* = 3.3 and β = 0.1 show the break in κ-continuation of the LC (IPB regime) that occurs when α is increased. Simulations also show that reducing *n* causes an increase in the critical value of α for the break in κ-continuation of LC. Decreasing *n* from 3.3 to 3.0 moves the critical value to between 45 and 50 compared to between 25 and 30 respectively in Fig. 2. We reproduce some of the interesting numerical results for β = 0.1, in experiment, as shown in Fig. 8. The circuit measurements of LC period and protein *B* LC amplitude as a function of κ were made for α = 46 and 60. The resulting continuous curves (green) and curves-with-break (blue) shown are in good qualitative agreement with the simulated behaviors in Figs. 2a and 2b. The IPB region is clear in Fig. 8 for α = 60. Figure 9 shows the LC for α = 46 for the two values κ = 14 and 19, demonstrating the decrease in both period and amplitude of LC. We were not able to increase κ high enough, in experiment, to observe the HB due to a practical limit on κ as discussed in Appendix S1.

**Figure 10:**
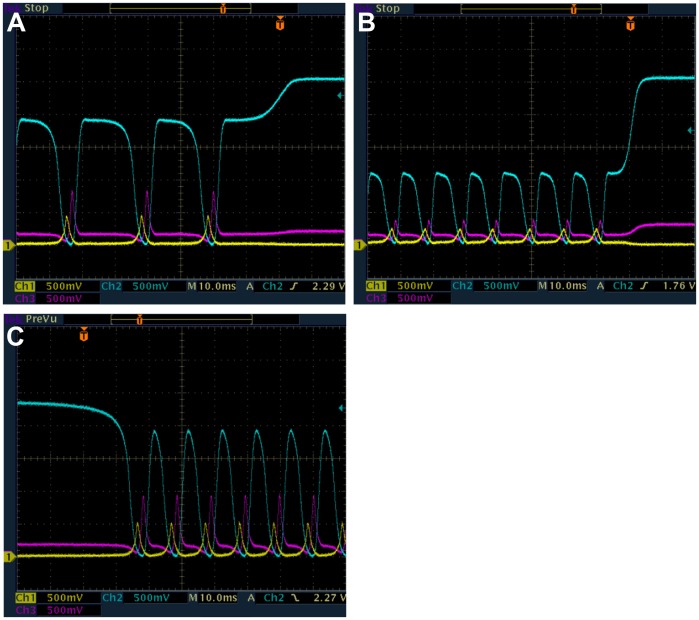
Measured time series from e-Rep circuit while slowly changing κ. β = 0.1 *n* = 3 α = 60, *k*_S1_ = 0.025, *k*_S0_ = 1, and η = 1 (protein *A*-yellow, *B*-blue and *C*-purple). (a) Increasing κ causes IPB at κ = 9.5. (b) Decreasing κ causes IPB at κ = 15.8. (c) Decreasing κ causes SS to LC transition at κ = 7.9. IPB in frames (a) and (b) are indicated by the increased width of protein *B* pulses relative to *A* and *C* pulses.

The break in κ-continuation of LC in Fig. 8 (blue) for β = 0.1, α = 60, *n* = 3.0 was investigated by slowly changing κ (increasing from low value as well as decreasing from high value) until transition of LC to SS was observed. These transitions as shown in Fig. 10 are the IPBs, occurring for increasing κ at 9.5 (Fig. 10a) and for decreasing κ at 15.8 (Fig. 10b). The hysteresis as predicted in Fig. 2 is also observed in Fig. 10 where the transition from SS to LC occurs for decreasing κ at 7.9 (Fig. 10c) compared to 9.5 for LC to SS. The range of κ for the coexistence of LC and SS due to hysteresis is clearly from 7.9 to 9.5. For β = 0.1, the only LC observed is period-1, in agreement with the predictions in Fig. 2.

**Figure 11:**
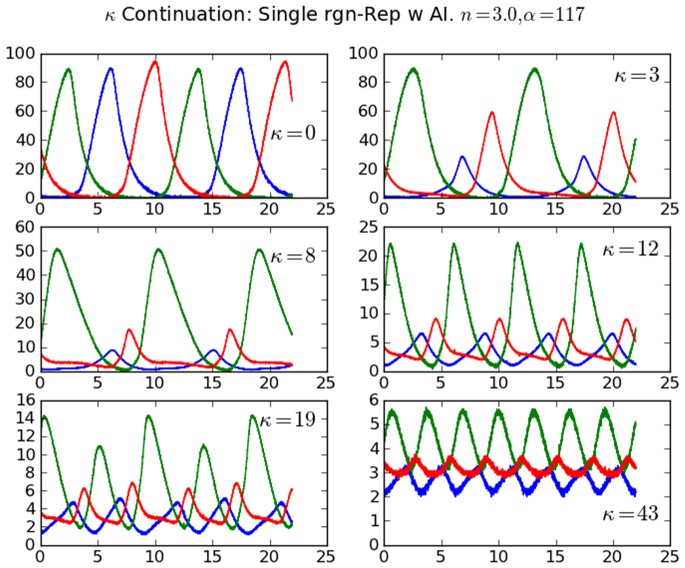
Measured time series from e-Rep circuit showing LC behavior for increasing QS strength κ. β = 1, *n* = 3, α = 117, *k*_S1_ = 0.025, *k*_S0_ = 1, and η = 1. Protein *A* (blue), *B* (green), *C* (red). LC is period-1 except for κ = 19 where it is period-2. Dimensionless time and concentration as used in the model (Eq. 1) are given on abscissa and ordinate for easy comparison to numerical predictions.

For β = 1, a forward and backward period-doubling of LC as shown by numerical simulations in Fig. 4 is realized in circuit measurements for the case β = 1, α = 117, n = 3. Figure 11 shows the evolution of the LC including period-1 and period-2. Plots of measured data are shown instead of oscilloscope images so that dimensionless values can be compared to predictions in Fig. 4. A steep decrease in amplitude of LC is apparent as κ increases from 0 to 12, as is the period-2 LC for κ = 19. The small amplitude LC for κ = 43 is close to the HB to SS. The lower red line in Fig. 4 for κ = 6 to 11 corresponds to a small peak that appears in the plateau shown in the κ = 8 case in Fig. 11.

**Figure 12:**
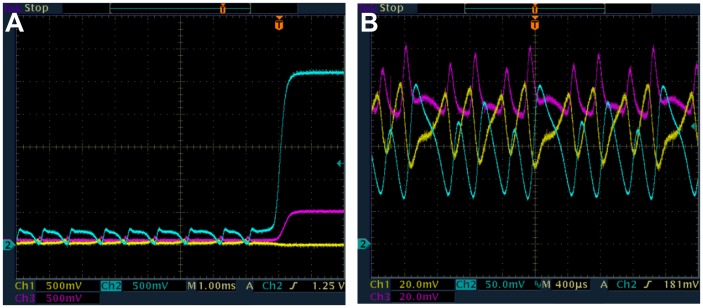
Measured time series from e-Rep circuit showing break in κ-continuity and period-4 oscillations. **β = 1, ***k*_S1_ = 0.025, *k*_S0_ = 1, and η = 1 (protein *A*-yellow, *B*-blue and *C*-purple).**** (a) IPB to High-*B*-SS occurring at κ≈26 for decreasing κ with n = 3, α = 138. (b) Period-4 LC for n = 3.4, α = 77. Note in panel (b) protein *B* is shown with 50 mV/div, *A* and *C* with 20 mV/div so peak-to-peak is about 170 mV for *B*, 50 mV for *A*, and 40 mV for *C*.

For β = 1 with larger α, a break in κ-continuation of the LC is observed as predicted in Fig. 3b similar to what is seen with β = 0.1. Figure 12a shows the IPB for decreasing κ from large values for β = 1, α = 138, n = 3. More complex dynamics predicted for larger *n* (Fig. 3b and 3c) including period-4 LC are seen (Fig. 12b) for β = 1, α = 77, n = 3.4.

### Noise Amplification Near IPB

**Figure 13:**
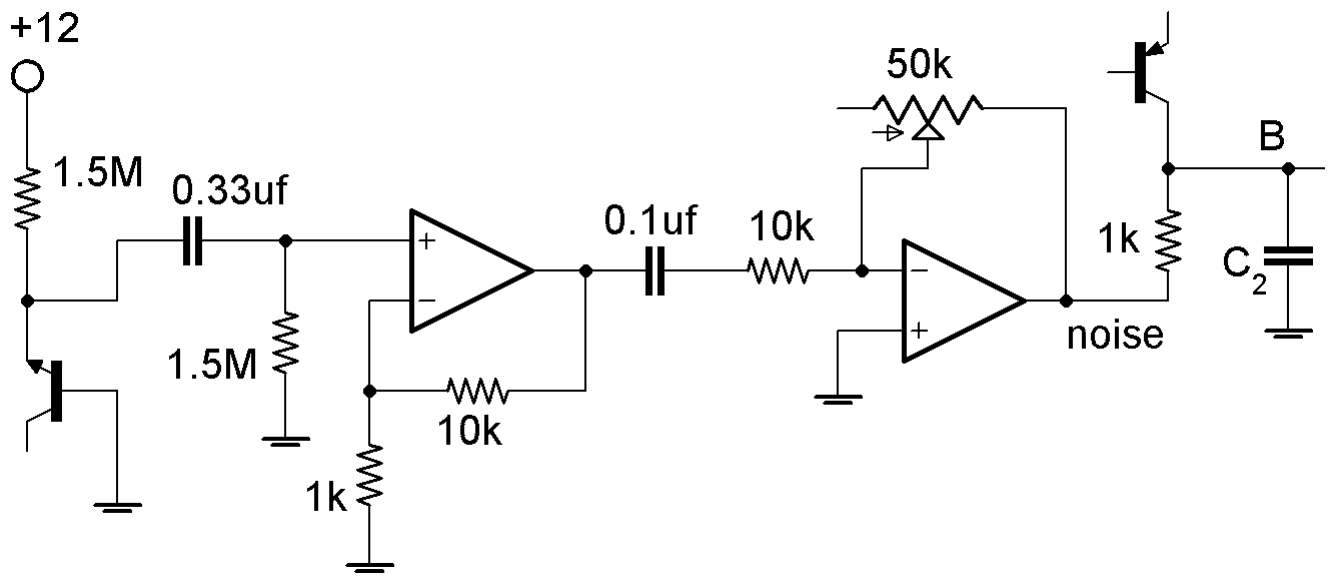
Noise circuit and its connection to protein ***B*** **in e-Rep circuit.** The source of noise is the reverse-biased base-emitter junction of the 2N3904 *npn* on left. The *npn*’s collector is unconnected. LF412 dual op-amp with +/−12 V supply.

A pre-bifurcation noise amplification [30] is predicted for the repressilator LC as *κ* approaches the IPB. We added noise to the circuit by connecting the voltage *V*_B_ (analog of protein *B*) to a noise source instead of to ground through the 1 kΩ resistor as shown in Fig. 13. The equation for the voltage *V*_B_ then becomes,
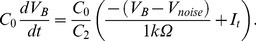
(5)where *I*_t_ is current from the transistor. The source of noise is the breakdown of the reverse biased base-emitter junction in the *npn* transistor in Fig. 13. The noise circuit is supplied by a well regulated ±12 V in order to avoid introducing AC noise.

The normalized noise is *x*_noise_ = *V*_noise_/*V*_th_ giving the equation for variable *B*

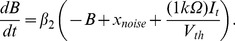
(6)


Ideally, the noise *x*_noise_ should have zero mean and an amplitude given by its standard deviation. Any dc component of the noise from the reverse biased *npn* is blocked by the coupling capacitors. Therefore the mean value of the noise is due solely to the nominal 1 mV offset error of the second op-amp. For example, with α = 60, then *V*_th_ = *I_max_R_C_*/*α* = (2.7 V)/60 = 45 mV, and the mean value of *x*_noise_ is 1 mV/45 mV ≈ 0.02. A similarly small value occurs for the minimal amplitude of *x*_noise_ due to a nominal 1 mV rms noise at the op-amp output. Fourier transforms of the noise show the frequency content to be uniform up to a cut-off around 300 kHz. Using the time-scale RC = 0.1 ms gives a dimensionless period of 1/(300 kHz×0.1 ms) = 0.033, which is much smaller than any of the LC periods. We conclude that the noise circuit provides a good approximation to zero mean random noise.

Fluctuations in LC period were measured for different noise amplitudes for three values of κ. The highest value, *κ* = 7.9, is close to the value for IPB when other parameters are *n* = 3, *α* = 60, *β*_i_ = 0.1, *k*_S1_ = 0.025, *S*_cr_ = 0.25. Results in Table 1 show the LC’s increased sensitivity to noise near the IPB. Far from the IPB (κ = 0) there is almost no effect on the LC period as noise is added. Close to the IPB (κ = 7.9) fluctuations of the LC’s period increase from 0.1 to 1.6 (standard deviation) when noise is added. The increase in period as κ approaches the IPB is also apparent.

**Table 1:** LC period (mean and standard deviation) as function of QS feedback and noise level.

	noise = 0.029	noise = 0.443	noise = 0.533	noise = 0.689
κ = 0	8.72±0.040	8.72±0.046	8.71±0.045	8.72±0.049
κ = 6.9	10.52±0.043	10.53±0.10	10.60±0.13	10.41±0.15
κ = 7.9	16.4±0.10	16.0±1.0	16.5±1.6	SS

The mean and standard deviation of e-Rep LC period was measured for different QS strengths κ and noise levels *x*_noise_. Period and noise are given in dimensionless quantities as defined in text. For LC far from IPB (κ = 0) the noise does not induce period fluctuations as evidenced by small change in standard deviation. For LC close to IPB (κ = 7.9) the noise causes large increase in LC period standard deviation, with fluctuations so large at *x*_noise_ = 0.689 that a transition to SS occurs. The increase in LC period with increasing κ due to IPB is apparent at all noise levels.

### Conclusions

Synthetic genetic oscillators are usually considered as prototypes of some natural genetic elements, or at least as motifs of genetic networks. Quorum sensing is a natural process of interaction or coupling between the oscillators. A 7-dimensional repressilator with quorum sensing feedback is the simplest genetic ring oscillator which demonstrates limit cycle oscillation over a very broad interval of control parameters. One of the possible motifs made from 3-genes [31] is shown in Fig. 1 and is our present candidate of interest. We made a reduction to a 4-dimensional model, in this work, that exhibits limit cycle behavior very similar to that of the original 7-dimensional repressilator. Additionally, quorum sensing is realized in the model as an additive regulatory function through an additional path in which protein *B* activates the production of protein *C* through an autoinducer. It is important that one protein (*B*) inhibits and activates, at the same time, the subsequent neighboring protein. This competition between inhibition and activation of the one element (*C*) in the ring of genes results in the coexistence of stationary and complex periodic regimes and opens new possibilities in the regulation of the repressilator.

The first manifestation of autoinducer production is the appearance of a stable steady state with high level of protein *B*. This steady state may be a single fixed point of the repressilator or may represent the high level of a genetic switch if the activator activity of autoinducer is large enough to allow the existence of a second stable steady state in which all proteins are at low level. The position of steady state does not depend on the time scales of Eq. (1) but oscillatory dynamics of the repressilator depends strongly on them.

For both fast and slow protein kinetics the limit cycle coexists with steady state, the cyclic behavior is suppressed by autoinducer via supercritical Andronov-Hopf bifurcation, and two infinite-period bifurcations exist when protein transcription (α) is increased enough to produce a break in the limit cycle’s κ-continuity. For slow protein kinetics (β’s = 0.1) the autoinducer feedback does not change the form of the limit cycle from period-1. This is not surprising because for small β the equation for autoinducer may be reduced resulting in a 3-dimensional system.

For identical time scales (β’s = 1) the equation for autoinducer may be considered as a delay mechanism in the appearance of activation of protein *C* production. It is expected that delay can stimulate complex oscillatory regimes. We show the cascades of period doubling bifurcations, the number of which along κ-axis is increased with α. However, these cascades do not result in chaos but end in period-halving and several infinite-period bifurcations.

Further we observed noise amplification in the form of increased fluctuations of limit cycle period (protein *B* concentration) near the infinite-period bifurcation in the presence of additive noise.

We present an electronic circuit model for a single repressilator with quorum sensing feedback that provides a useful tool which is used in combination with numerical simulations to demonstrate rich dynamics: steady states, switch, limit cycles, infinite-period bifurcations, period doublings. These results for an isolated repressilator under the influence of QS self-feedback should be beneficial for understanding communication between coupled repressilators via QS. In addition, we demonstrate that the electronic analog may be used as an effective test bed for the studies of regulations in genetic networks, and more generally for studies of particular network topologies potentially leading to reverse engineering such as the design of useful electronic devices. Recently we have used the electronic gene in Fig. 5 for investigation of logical stochastic resonance in a 2-gene network [29].

## Supporting Information

Figure S1 **Circuit simulation of inhibitory Hill function for** ***n*** **= 3.** Output is the measured normalized current (blue) from the transistor in the single gene circuit (Fig. 5). *V* is the inhibition input voltage and *V*_th_ accounts for the inhibitor binding affinity. Analytic Hill function 1/(1+ *x* ^n^) (red).
(TIF)

Figure S2 **Circuit simulation of quorum sensing S-function.** In the QS circuit (Fig. 7) the *pnp* transistor produces S-function activated protein C current which goes to the protein *C* capacitor voltage. Measured S-function circuit current (blue), piece-wise continuous linear model min(S/(1+S_cr_),1) (green), and S/(1+S) (red). *V* corresponds to the concentration S of AI and V_sth_ accounts for the binding affinity of AI activator. S_cr_ = 0.25.
(TIF)

Appendix S1 **Details of circuit analysis.**
(DOC)
