# Supplementary figures and images for: Electronic Implementation of a Repressilator with Quorum Sensing Feedback
Source: PLoS One. 2013 May 2;8(5):e62997. doi: 10.1371/journal.pone.0062997 (PMC3642084; doi:10.1371/journal.pone.0062997)

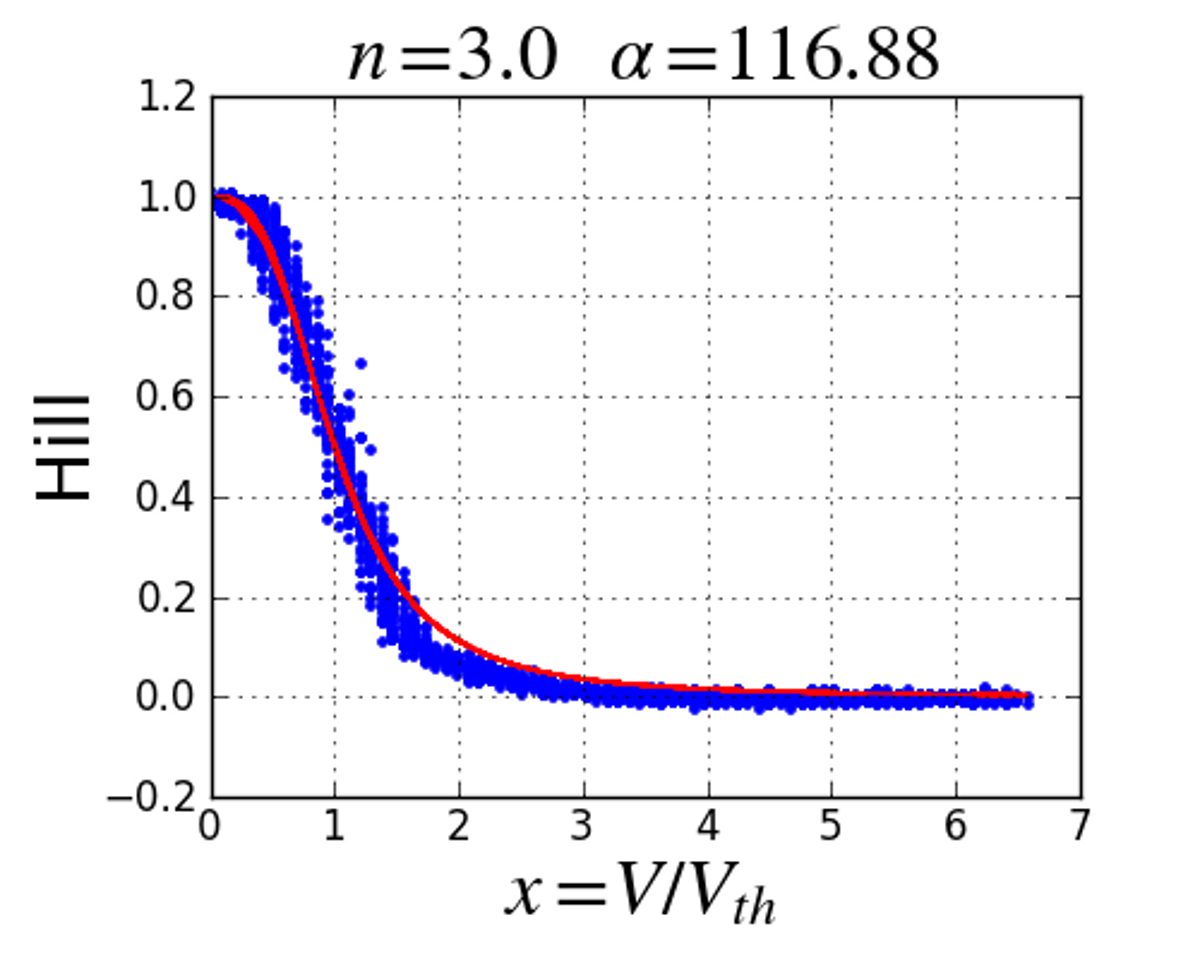

Supplement: Figure S1 — Circuit simulation of inhibitory Hill function for n = 3. Output is the measured normalized current (blue) from the transistor in the single gene circuit (Fig. 5). V is the inhibition input voltage and V th accounts for the inhibitor binding affinity. Analytic Hill function 1/(1+ x n) (red). (TIF) [file pone.0062997.s001.tif]

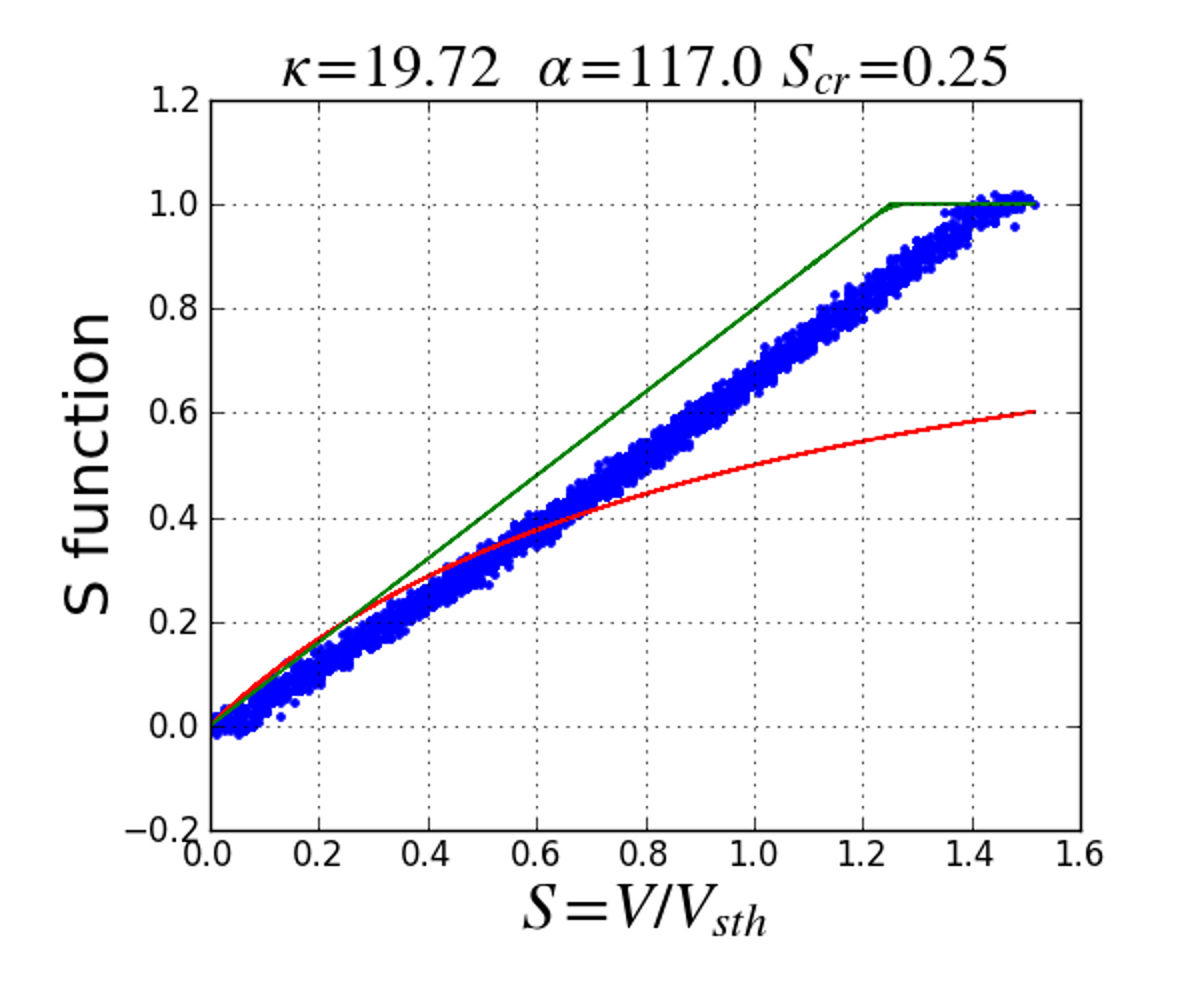

Supplement: Figure S2 — Circuit simulation of quorum sensing S-function. In the QS circuit (Fig. 7) the pnp transistor produces S-function activated protein C current which goes to the protein C capacitor voltage. Measured S-function circuit current (blue), piece-wise continuous linear model min(S/(1+Scr),1) (green), and S/(1+S) (red). V corresponds to the concentration S of AI and Vsth accounts for the binding affinity of AI activator. Scr = 0.25. (TIF) [file pone.0062997.s002.tif]
