# Supplementary material for: Electronic Implementation of a Repressilator with Quorum Sensing Feedback
Source: PLoS One. 2013 May 2;8(5):e62997. doi: 10.1371/journal.pone.0062997 (PMC3642084; doi:10.1371/journal.pone.0062997)
Supplement: Appendix S1 — Details of circuit analysis. (DOC) [file pone.0062997.s003.doc]

**Appendix S1**

*Circuit with Improved Hill Function*

We present a modified version of the e-Rep circuit here that produces a better approximation of the Hill function kinetics than the circuit in Ref. 28. Gene inhibition is modeled by the Hill function

(A1)

where *x* is a dimensionless concentration of the inhibitory protein. Parameter *V_th_* accounts for the inhibitor’s equilibrium binding constant and is used to convert voltage to concentration *x_i_* = *V_i_*/*V_th_*.

The circuit in Ref. 28 used a piecewise linear approximation of the Hill function. Here, we use three diodes (Fig. 5) in the feedback of op-amp U2 to add a positive curvature to the decay. As in Ref. 28 the op-amp U2 has different gains, *G_-2_* when *V_i-1_* < *V_cth_*, and *G_+2_* when *V_i-1_* > *V_cth_*. For component values in the figure the subtraction op-amp U1 has *G*_1_ = -6.8, and inverting op-amp U2 has *G*_-2_ = -20 and *G*_+2_ is an amplitude-dependent diminishing gain due to the diodes.

The gene inhibition in the circuit corresponds to *V_i_*_-1_ surpassing *V_cth_*, which causes the output of U2 to go positive and thereby turns off the *pnp* transistor (protein expression). The maximum output voltage of U2 is about 1.8 V when the three diodes are fully conducting in their forward biased state. The resistors *R*_b1_ and *R*_b2_ are chosen such that an output voltage at U2 of 1.8 V causes a drop of (0.4/2.6)(5 − 1.8) = 0.49 V across *R*_b1_ which is small enough so that the transistor current is essentially zero. In the circuit, the maximal protein expression corresponds to *V_i_*_-1_ = 0 which results in U2 output going negative with a limit at the lower saturation level *V_-sat_* = -3.5 V for the dual op-amp LF412 supplied by +/-5 V. We assume that the gain *G*_1_*G*_-2_ is large enough so that the output of U2 reaches *V*_-sat_ when *V*_i-1_ = 0. Later we determine a practical restriction on Hill coefficient *n* imposed by this assumption.

Here we predict the transistor current (Fig. 5) when the output of U2 varies between -3.5 and 1.8 V. The voltage across R_b1_ is where the fraction *f* = 0.4/2.6 = 0.154 is the voltage divider gain, Δ*V* = (*V_i_*_-1_ − *V_cth_*), and *G* is the overall gain of the 2 op-amps. The current through *R*_E_ which flows out from the transistor’s collector is

(A2)

where the 0.55 volts approximates the emitter-base voltage drop (0.55 is an overestimate at low currents and an underestimate at high currents.) Maximal protein expression occurs for *V_i_*_-1_ = 0 (no inhibition) and thus U2 output *G*Δ*V* = *V_-sat_* giving maximum current

(A3)

where 0.6 volts is a better approximation for the emitter-base voltage drop than 0.55 when at maximum current. For our circuit values the *I*_max_ ≈ 3 mA. In the circuit, the Hill function corresponds to the normalized current

. (A4)

As in Ref. 28, we approximate the Hill function by setting the slope of the normalized current equal to the slope of the Hill function *dH*/*dx* at *x* = 1. Using Δ*V* = *V_th_*(*x_i_*_-1_ − *V_cth_*/*V_th_*), gain *G* = *G_1_G_-2_* for *x* = 1, and setting the slopes equal gives the condition for the Hill coefficient *n*

. (A5)

For our circuit values for *f* and *V_-sat_*, and using *V_th_* = *I_max_R_C_*/*α* with *I_max_R_C_* = 2.7 V (*R*_C_ = 0.9kΩ accounts for the 10kΩ input to U1), we determine,

. (A6)

Next we find the relationship between binding constant term *V_th_* and the circuit parameter *V_cth_*. At *x* = 1 the Hill function has a value of 0.5. The corresponding condition for the circuit is that the normalized transistor current be 0.5 when *V_i_*_-1_ = *V_th_*. Setting Eq. (A4) to 0.5, letting Δ*V* = (*V_th_* − *V_cth_*), and solving gives *V_cth_* = *V_th_* + 0.87/(*G_1_G_-2_*) ≈ *V_th_* + 1/(*G_1_G_-2_*). For the circuit in Fig. 5, *G*_1_*G*_-2_ = (-6.8)×(-20) so that *V_cth_* = *V_th_* + 7.4 mV.

The assumption that the output of op-amp U2 is saturated at *V*_-sat_ when *V*_i-1_ = 0 (no inhibition) means that *G*_1_*G*_-2_*V*_cth_ > 3.5 V. Using the relations between *V*_cth_ and *V*_th_, between *V*_th_ and α, and between *G*_1_*G*_-2_ and *n*α, we find the restriction on the Hill coefficient *n* > 2.35(-*V*_-sat_ − 1)/(*i*_max_*R*) = 2.35(3.5 − 1)/2.7 = 2.2.

In U2’s feedback the value of the resistor in parallel with the two series diodes is varied to match the transistor current’s decay to that of the Hill function. For small values of *n* the Hill decay extends to larger *x* values meaning a larger input *V*_i-1_ is required to turn off the transistor. Reducing the resistor value has the desired effect since a larger *V*_i-1_ is needed for U2 to produce enough current in the resistor to bring the diodes into forward biased conduction. For the case of large *n*, increasing the resistor allows lower currents to bring the three diodes into conduction thereby saturating U2’s output at 1.8 V. It is convenient to use a 5kΩ trim-pot and to determine the optimal value in a test circuit. For *n* = 3 we find 3.3kΩ is a good value as shown by the measured response and the mathematical Hill function in Figure S1.

*Quorum Sensing Circuitry*

Here we find the relations between model parameters (κ,S_cr_) and circuit parameters for the quorum sensing feedback. Figure 7 shows the quorum sensing circuitry and its connection to the e-Rep voltages corresponding to proteins *B* and *C*. The current labeled *κS*/(1+*S*) corresponds to the AI feedback production of protein *C* in Eq. (1c). We approximate the *S*-dependence by a piece-wise linear model min(S/(1+S_cr_),1) which is achieved by the op-amp driving the combination of *npn* and *pnp* transistors. The transistors are both slightly on when *V*_S_ = 0 such that the current sourced by the *pnp* is sunk by the *npn*. This allows the *pnp* to be in a nearly linear range of operation even when *V*_S_ = 0. As *V*_S_ increases, the *npn* shuts off and all the *pnp* current exits the QS circuitry through the diode. Circuit analysis predicts that the current leaving the QS circuitry is approximately,

(A7)

where for our component values the voltage divider fraction is *f*_S_ = (1.33/11.3) = 0.117 and the output of the op-amp is *G*_S_*V*_S_ where *G*_S_ = -(*R_f_*/*R*_S2_). The maximum current occurs for large *V*_S_ making *G*_S_*V*_S_ = *V*_-sat_. With *V*_-sat_ = -3.5 V, the predicted maximum current is *I*_Smax_ = 0.4/*R*_κ_, while measurement finds *I*_Smax_ = 0.35/*R*_κ_. The relation between κ and *I*_Smax_ is the same as between α and *I*_max_ for e-Rep protein expression, giving

. (A8)

*R*_C_ and *V*_th_ are determined by the e-Rep, so *R*_κ_ is a potentiometer used to set κ. A practical limit on the maximum value for κ is given by the restriction R_κ_ > 250Ω so that the dynamic resistance of the transistor’s base-emitter junction can be neglected. Using *V*_th_ = *I*_max_*R*_C_/α gives restriction κ < α/2.

The normalized current which represents the S-function is *I_S_/I_Smax_* = -0.334*G_S_V_Sth_S.* In order to match the normalized current to the piece-wise linear S-function min(S/(1+S_cr_),1) we set their slopes equal giving

. (A9)

We choose *S*_cross_ = 0.25 which gives the relation *G*_S_*V*_sth_ = –2.4, thereby determining *G*_S_ for a specified value of *V_Sth_*. The value of *G*_S_ determines *R*_f_ in the op-amp’s feedback. For example if *V*_th_ = 40 mV, *k*_S1_ = 0.025, and resistors *R*_S1_ = 100 kΩ and *R*_S2_ = 6.8 kΩ, then *V*_sth_ = 109 mV and *G*_S_ = -*R*_f_/6.8k = -22, giving *R*_f_ = 150 kΩ.

Figure S2 shows the piece-wise linear approximation, measured normalized current, and the function *S*/(1+*S*) for *S*_cross_ = 0.25.

Initial conditions for protein voltages are set by using an analog switch (4066) to momentarily connect the capacitor voltages *V*_1_, *V*_2_, and *V*_3_ to set voltage sources.
